# Function of Macrophages in Disease: Current Understanding on Molecular Mechanisms

**DOI:** 10.3389/fimmu.2021.620510

**Published:** 2021-03-08

**Authors:** Chunye Zhang, Ming Yang, Aaron C. Ericsson

**Affiliations:** ^1^Department of Veterinary Pathobiology, University of Missouri, Columbia, MO, United States; ^2^Department of Surgery, University of Missouri, Columbia, MO, United States; ^3^Department of Veterinary Pathobiology, University of Missouri Metagenomics Center, University of Missouri, Columbia, MO, United States; ^4^Department of Veterinary Pathobiology, University of Missouri Mutant Mouse Resource and Research Center, Columbia, MO, United States

**Keywords:** macrophage, phenotype, molecules, diagnostic marker, therapy

## Abstract

Tissue-resident macrophages (TRMs) are heterogeneous populations originating either from monocytes or embryonic progenitors, and distribute in lymphoid and non-lymphoid tissues. TRMs play diverse roles in many physiological processes, including metabolic function, clearance of cellular debris, and tissue remodeling and defense. Macrophages can be polarized to different functional phenotypes depending on their origin and tissue microenvironment. Specific macrophage subpopulations are associated with disease progression. In studies of fate-mapping and single-cell RNA sequencing methodologies, several critical molecules have been identified to induce the change of macrophage function. These molecules are potential markers for diagnosis and selective targets for novel macrophage-mediated treatment. In this review, we discuss some of the recent findings regarding less-known molecules and new functions of well-known molecules. Understanding the mechanisms of these molecules in macrophages has the potential to yield new macrophage-mediated treatments or diagnostic approaches to disease.

## Introduction

Macrophages are an essential component of the innate immune system, with a wide distribution in lymphoid and non-lymphoid tissues throughout the body. Macrophages were initially known to arise from circulating blood monocytes that continuously migrate to different tissues and differentiate into macrophages (1). Although the origin of adult tissue-resident macrophages (TRMs) is still not totally understood, it is now recognized that TRMs are derived from diverse progenitors, including embryonic origin and monocyte progenitors. Some fate-mapping studies have demonstrated that major TRMs in mice, such as liver resident Kupffer cells (KCs) and lung alveolar, splenic, and peritoneal macrophages, are developed before birth and can maintain themselves independent of circulating blood monocytes (2, 3). Further study also demonstrated that those TRMs such as KCs in mouse fetal liver originate from yolk sac erythro-myeloid progenitors (EMPs), which are distinct from adult hematopoietic stem cells (HSCs) (4). In contrast, other studies showed that some TRMs such as adult cardiac and skeletal muscle macrophages are derived both from yolk-sac and fetal monocyte progenitors (5, 6), and embryonically developed TRMs can be replaced by blood monocytes.

Macrophages have pivotal functions in homeostasis and many physiological processes beyond innate immunity, including metabolic function (7), clearance of cellular debris (8), tissue repair and remodeling (9). In pathogenic conditions, TRMs can be replaced and joined by recruited monocyte-derived macrophages to orchestrate an immune response (10). Current studies using single-cell RNA-sequencing (scRNA-seq) have revealed the presence of multiple macrophage subsets with distinct functions in various tissues (11). For example, Remmerie et al. (12) reported that in fatty liver, hepatic resident KCs were destroyed and replaced by bone marrow-derived macrophages, and those recruited macrophages comprise two subsets resembling either KCs or lipid-associated macrophages expressing Osteopontin. Moreover, macrophage function and phenotype are impacted by various factors in physiological and pathological conditions, such as diet (13) and cytokines (14). The alteration of macrophage phenotype or polarization is associated with distinct gene expression profiles (15).

Herein, we summarize the latest findings of new macrophage markers in different diseases. Firstly, the phenotypes of macrophages are briefly discussed. Secondly, some macrophage-associated molecules are selected to discuss their important roles in diseases, especially with regard to newly identified functions. Then, the potential application of some molecules as diagnostic markers or therapeutic targets for treatment is reviewed. Finally, the methods that are applied to deplete macrophages are discussed.

## Macrophage Phenotypes

**Figure 1:**
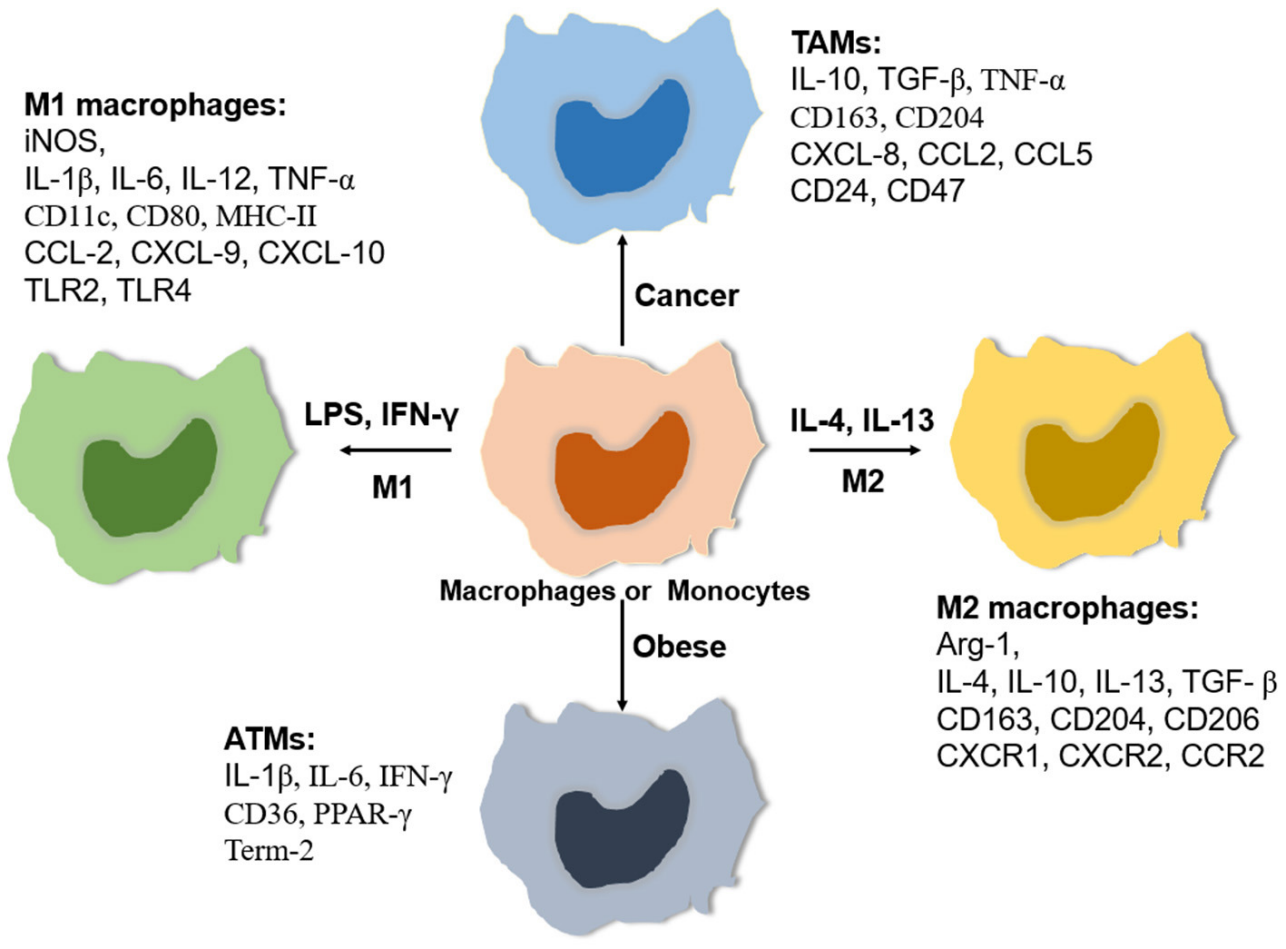
Relative expressing gene profiles. Macrophages are classically polarized into pro-inflammatory macrophages (M1) induced by LPS/ LPS plus IFN-γ or activated into anti-inflammatory macrophages (M2) induced by IL-4/IL-13. In addition, macrophages can adapt special tissue microenvironments to polarize specific phenotypes such as tumor-associated macrophages (TAMs) and adipose tissue macrophages (ATMs). Each phenotype of macrophages has a relatively specific expression of some cytokines, chemokines, Toll-like receptors (TLRs), and matrix metalloproteinases (MMPs).

Macrophages are very plastic cells with different phenotypes and functions (Figure 1), which are impacted both by their origin and resident tissue microenvironment. Broadly, macrophages can be activated into two distinct subsets based on the M1/M2 paradigm, classically activated or M1 macrophages and alternatively activated or M2 macrophages (16). M1 macrophages are polarized *in vitro* by Th1 cytokines such as colony-stimulating factor (GM-CSF), tumor necrosis factor α (TNF-α), and interferon-γ (IFN-γ) alone or together with lipopolysaccharide (LPS) from bacteria. M1 macrophages express pro-inflammatory cytokines such as interleukin-1β (IL-1β), IL-6, IL-12, IL-23, and TNF-α (17). In contrast, M2 macrophages are polarized by Th2 cytokines such as IL-4 and IL-13 and produce anti-inflammatory cytokines such as IL-10 and transforming growth factor beta (TGF-β) (18).

Recent studies have revealed that the M1/M2 paradigm is not sufficient to encompass all states of macrophage activation. Macrophages have the ability to change their polarization in response to different stimuli. For example, macrophage phenotype changes during tissue repair, switching from pro-inflammatory phenotype (M1-like) to an anti-inflammatory phenotype (M2-like) (19). In addition, different polarization and activation markers of M1- and M2-like macrophages can coexist in tissues (20). For instance, a high percentage of circulating macrophages expressing both M1 (CD80, CD86, and TLR4) and M2 surface markers (CD204, CD163, and CD206) was shown in human patients with interstitial lung disease (21). With the analysis of the transcriptomic profiles of macrophages, Liu et al. (22) reported that polarized M1- or M2-like macrophages driven by cytokines can be subsequently repolarized to another phenotype with little or no memory of polarization history. Therefore, the *in vivo* phenotype and function of macrophages remains to be defined under specific tissue microenvironments.

Phenotypic change and functional polarization of macrophages are accompanied by a change in cellular metabolism, as M1-like macrophages primarily rely on glycolysis, whereas M2-like macrophages rely on oxidative phosphorylation (23). Parallel analysis of macrophage metabolic and transcriptional profiles also indicates that metabolic reprogramming impacts macrophage polarization or activation (24). For example, inhibition of aspartate-aminotransferase and N-glycosylation interfere with M1 and M2 macrophage polarization, respectively. Factors that affect macrophage metabolism may disrupt M1/M2 homeostasis. Hill et al. (25) reported that multiple distinct populations of adipose tissue-associated macrophages (ATMs) are present in adipose tissues in mice and humans, existing with unique transcriptomes, chromatin landscapes, and functions. Similarly, pro-inflammatory ATMs in obese mice or humans can express additional markers of metabolic activation distinct from the classical markers of activation (26).

In addition to the abilities of engulfing and digesting foreign pathogens and cellular debris, macrophages can clear away tumor cells. Tumor-associated macrophages (TAMs) are particularly abundant immune cells in cancer and exert strong influences on tumor initiation, progression, and metastasis (27, 28). Besides, TAMs can secrete different cytokines such as IL-10 and transforming growth factor-β (TGF-β) to suppress T cell-dependent antitumor function (29). TAMs can be polarized into pro-inflammatory (M1-like) phenotype and anti-inflammatory (M2-like) phenotype under the stimuli of different tumor microenvironments, with the majority of TAMs functioning as M2-like macrophages (30). Notably, differentially polarized TAMs may exert opposite effects on tumor development. For example, high numbers of infiltrating M2-like macrophages in patients with gastric cancer (GC) were associated with a low rate of overall survival (OS), while an elevated number of M1-like macrophages was associated with better OS (31). Using scRNA-seq data and cell trajectory analysis, Landry et al. (32) reported that core TAMs evolve toward a pro-inflammatory state in human glioblastoma, while peripheral TAMs develop an anti-inflammatory phenotype. Therefore, an accurate understanding of the function of TAMs is likely to advance cancer immunotherapy.

## Important Molecules Mediating Macrophage Function

Currently, scRNA-seq is a critical tool to investigate macrophage heterogeneity and function. For example, transcriptional profiling analysis showed that there are five clusters of alveolar macrophages (AMs) across homeostasis, acute inflammation, and resolving inflammation, all expressing macrophage-specific markers, such as CD68 and Lgals3 (Galectin-3), Among them, two clusters express tissue-resident airspace macrophage marker genes, such as Mrc1 (CD206) and Itgax (CD11c), and another three clusters with significant upregulation of recruited AM marker genes, such as CD14, Ly6c1 (Ly6c), and Sell (L-selectin) (33). In addition, resident AMs show higher expression of M2-like macrophage genes and two clusters of recruited AMs have higher expression of M1-like macrophage genes, while the last cluster of recruited AMs exhibit relatively low expression of both M1-like and M2-like gene expression profiles. Overall, these data further indicate that the M1/M2 paradigm is not sufficient for classifying macrophage polarization. In this section, we highlight some important genes found in macrophage function and transcriptional profiling studies.

## CD163

The hemoglobin scavenger receptor (CD163) is a macrophage-specific protein (34), highly expressed on TRMs but modestly expressed on monocyte-derived macrophages. The expression of CD163 was up-regulated on human blood monocytes following stimulation with macrophage colony-stimulating factor (M-CSF), and down-regulated following stimulation with GM-CSF and IL-4 (35). In addition, CD163 expression was also suppressed by pro-inflammatory molecules and cytokines such as LPS, IFN-γ, and TNF-α, and upregulated by anti-inflammatory cytokine IL-10, indicating CD163 is expressed on macrophages with the anti-inflammatory phenotype (35). Recent findings show that CD163^+^ macrophages promote tumor progression. Shiraishi et al. (36) reported that CD163^+^ macrophages were associated with decreased overall survival of patients with pleomorphic sarcoma. Silencing CD163 abrogated macrophage-induced tumor cell proliferation in co-cultured cells of human monocyte-derived macrophages and leiomyosarcoma and myxofibrosarcoma cell lines. *In vivo* assays demonstrated the growth of sarcoma was significantly inhibited in CD163-deficient mice compared to wild-type mice, which was associated with the production of IL-6. In breast cancer, CD163-expressing TAMs resembling an M2-like phenotype accumulated in the tumor microenvironment and were associated with poor clinical outcomes (37). A similar difference is also found in colorectal tumors (CRC). The anti-inflammatory CD163^+^ macrophages (M2-like) were more prevalent in advanced tumor stage and exclusively located in invasive tumor front, whereas the CD80^+^ macrophages (M1-like) were predominant in less invasive tumors and predominantly distributed in tumor-adjacent normal mucosa (38). Moreover, a low CD80/CD163 ratio was associated with decreased overall survival in patients with CRC (39).

## LYVE-1

Lymphatic vessel hyaluronan receptor-1 (LYVE-1), the major receptor of hyaluronan (HA) in lymphatic vessel endothelial cells, is closely related to the leukocyte HA receptor CD44 (40), mediating the trafficking of leukocytes, including macrophages. Lim et al. (41) reported that LYVE-1^+^ macrophages line murine and human blood vessels, modulated collagen production in smooth muscle cells to maintain arterial wall homeostasis. Another study also showed LYVE-1^+^ cells expressing macrophage markers CD68 or CD169 that co-localized with collagen fibers in rat meninges, and some LYVE-1^+^ cells had intracellular collagen (42). Dollt et al. (43) also reported that extracellular domains of LYVE protein from M2-like macrophages significantly inhibited human and murine melanoma cell proliferation by acting as a receptor for low-molecular weight HA. In addition, the LYVE-1^+^ macrophages have been shown to play critical roles in tissue remodeling (44) and murine eyes (45).

## MerTK

Synovial tissue macrophages (STMs) play critical roles in autoimmune diseases including rheumatoid arthritis (RA) (46). With the analysis of integrated scRNA-seq, deep-phenotypic, spatial and functional data, Alivernini et al. (47) found that synovial tissue macrophages (STMs) can be broadly classified into two populations, MerTK^−^CD206^−^ STMs and MerTK^+^CD206^+^ STMs. MerTK^−^CD206^−^ STMs produce pro-inflammatory cytokines and alarmins and induce inflammatory responses in synovial fibroblasts, while MerTK^+^CD206^+^ STMs from patients with RA in sustained disease remission produce lipid mediators that resolve inflammation and induce a repair phenotype of fibroblast-like synoviocytes. Also, they found that two STM subpopulations (MerTK^+^TREM2^high^ and MerTK^+^LYVE1^+^) with unique remission transcriptomic signatures, enriched in negative regulators of inflammation (47). Kuo et al. (48) also identified a subset of inflammatory macrophages expressing heparin-binding EGF-like growth factor in RA joints, which altered synovial fibroblast gene expression profile (e.g., IL-33) via up-regulation of epidermal growth factor receptor (EGFR) response and increased their invasiveness (47, 48). MerTK as a key efferocytosis receptor, is primarily expressed by CD11b^+^ F4/80^+^ large peritoneal macrophages (LPMs). At steady-state, MerTK-deficient LPMs exhibit significantly increased pro-inflammatory cytokine expression, when under stimulation of apoptotic cells, MerTK^−/−^ LPMs increased gene expression of cell death and apoptosis (49).

## Siglecs

Siglecs (sialic acid-binding immunoglobulin-type lectins) are transmembrane surface proteins found primarily on hematopoietic cells, consisting of intracellular tyrosine motifs involved in cell signaling (50). Siglecs predominantly recognize sialic acid residues of glycoproteins on the cell membrane. Fourteen of 15 known Siglecs are present in humans, namely Siglec-1 to 15 except Siglec-13 which is present in nonhuman primates. Siglecs on macrophages or monocytes play critical roles in many different diseases, as described in Table 1. Siglecs appear to have predominantly pro- or anti-inflammatory functions in macrophages. For example, Siglec-1, a receptor on monocytes/macrophages, plays a central role in the pathogenesis of congenital heart block and contributes to IFN stimulation (51). Plasma concentrations of Siglec-1 are also strongly correlated with type I interferon-regulated gene expression in systemic lupus erythematosus (SLE) patients (75), suggesting a pro-inflammatory influence.

**Table 1:** The role of Siglecs in macrophage function.

**Siglecs**	**Diseases**	**Function**	**References**
Siglec-1 (CD169)	Congenital heart block (CHB)	The expression of IFN and type I IFN-stimulated genes, including Siglec-1, a receptor on monocytes/macrophages, play an important role in the pathogenesis of congenital heart block.	(51)
Siglec-2 (CD22)	Aging brain	CD22 (siglec-2) mediated the anti-phagocytic effect and inhibition of CD22 promoted the clearance of myelin debris, amyloid-β oligomers, and α-synuclein fibrils *in vivo*. A long-term CNS-delivery of a CD22 blocking antibody activated microglia, resulting in the improvement of cognitive function in aged mice.	(52)
Siglec-3 (CD33)	Alzheimer's disease	Deletion of hCD33 in macrophage cell lines U937 and THP-1 increased cargo uptake *in vitro*. In addition, transgenic mice expressing hCD33 in the microglial cell lineage showed inhibited cargo uptake in primary microglia.	(53)
Siglec-4 (Myelin-associated Glycoprotein, or MAG)	CNS pathology	Myelin-associated glycoprotein (MAG), a minor constituent of central and peripheral nervous system myelin, binds to gangliosides GD1a and GT1b, prominent molecules on the axon surface, mediating axon stability in the central nervous system and peripheral nervous system.	(54)
Siglec-5 (CD170)	Asthma	Siglec-5 expression was significantly increased in patients receiving inhaled corticosteroids, exerting a beneficial effect. Double staining of cells indicated that Siglec-5 was expressed in monocyte/macrophages and neutrophils, but not in lymphocytes. Exposure to the sialic acid-expressing human bacterial pathogen group B Streptococcus (GBS), Siglec-5 negatively regulates inflammation.	(55, 56)
Siglec-6 (CDw327)	Colorectal cancer (CRC)	The siglec-6 expression on mast cells is involved in their function in the tumor microenvironment of CRC, but no evidence shown in macrophages.	(57)
Siglec-7 (CDw328)	Non-alcoholic fatty liver disease (NAFLD)	Serum Siglec-7 could serve as an independent marker for advanced liver fibrosis in patients with NAFLD.	(58)
Siglec-8 (Siglec-F in mouse)	Lung disease	Therapeutic targeting of Siglec-8 has the potential to impact blood as well as lung eosinophils, which may be associated with an improved outcome in eosinophilic lung diseases.	(59)
Siglec-9 (CD329 and Siglec-E in mouse)	Sepsis	Blockade Siglec-9 induced inflammation by anti-Siglec-9 Fab fragment (hS9-Fab03) is a potential therapeutic agent for sepsis.	(60)
Siglec-10 (Siglec-G in mouse)	Ovarian and breast cancers	Blockade of CD24-Siglec-10 signaling is a potential therapeutic strategy for breast and ovarian cancer immunotherapy.	(61)
Siglec-11	Neural disease	The polysialic acid (polySia) with an average degree of polymerization 20 (avDP20) neutralized the LPS-triggered increase in macrophage phagocytosis, by interacting with SIGLEC-11.	(62)
Siglec-12 (Siglec-like molecule-1)	Prostate cancer	The stable expression of Siglec-12 enhanced prostate cancer cell growth in nude mice. Anti-Siglec-12 monoclonal antibodies were internalized by Siglec-12-expressing prostate carcinoma cells, providing a target.	(63)
Siglec-14	Bacteria	Siglec-14 enhances NLRP3 inflammasome activation in macrophage, in response to known inflammasome activators or the sialic acid-expressing human bacterial pathogen GBS.	(56)
Siglec-15(Siglec-H in mouse)	Cancer	Siglec-15 on macrophages may contribute to tumor progression by targeting the sialyl-Tn (sTn) antigen, a tumor-associated glycan structure, which modulating TGF-β secretion in tumor microenvironments.	(64)

In the context of nonalcoholic fatty liver disease (NAFLD) and nonalcoholic steatohepatitis (NASH), liver Kupffer cells and recruited monocyte-derived macrophages play pivotal roles in the pathophysiology of NAFLD and NASH (76). In NAFLD patients, Siglec-7 was mostly expressed in hepatic CCR2^+^ macrophages, in contrast, its expression was much weaker in resident macrophages (58). In addition, soluble Siglec-7 was shown highly produced in monocyte-derived macrophages and serum sSiglec-7 can be used as an independent marker for NAFLD in patients with advanced liver fibrosis (58).

Siglec-9, as a mediator of inflammation, is a potential target for the treatment of sepsis. Pretreatment with a human anti-Siglec-9 Fab fragment attenuated LPS-induced pro-inflammatory cytokines TNF-α, IL-6, and IL-1β production in human peripheral blood mononuclear cell (PBMC)-derived macrophages and human THP-1-differentiated macrophages (60). Following exposure to the sialic acid-expressing human bacterial pathogen group B *Streptococcus* (GBS), Siglec-14, as a positive regulator of NLRP3 inflammasome activation on macrophages, resulted in the release of the pro-inflammatory cytokine IL-1β. Mononuclear phagocytes are also attractive drug delivery vehicles for novel cancer treatment owing to their cancerous tissue-accumulating nature. Given Siglecs are highly expressed in TAMs and peripheral blood monocytes, sialic acid-conjugated liposomes have been applied to deliver tumor-targeting drugs (77). Moreover, other Siglecs including Siglec-2 (52), Siglec-3 (53), Siglec-4 (54), Siglec-5 (55, 56), Siglec-6 (57), Siglec-8 (59), Siglec-10 (61), Siglec-11 (62), Siglec-12 (63), Siglec-15 (64) also play important roles in diseases as listed in Table 1.

## SIRPα

CD47, known as the “don't-eat-me” signal, is commonly overexpressed on the surface of tumor cells (78, 79). Signal regulatory protein α (SIRPα) on macrophages serves as a receptor of CD47 to transduce CD47/SIRPα axis mediated inhibitory function of macrophage phagocytosis of tumor cells (80). Blockade of CD47-SIRPα signaling is a strategy to promote macrophage phagocytosis of many cancer cells, which is discussed in the therapeutic section. A number of preclinical and clinical investigations are underway to target the CD47/SIRPα axis for cancer therapy, including examination of the synergistic effect with other anti-tumor agents such as rituximab (anti-CD20 antibody), cetuximab (an inhibitor of EGFR), and trastuzumab (an inhibitor of human epidermal growth factor receptor 2, HER2) (81).

## TREM2

The prevalence of obesity has reached epidemic levels, and over 44% of adults are overweight worldwide. Obesity substantially increases the risk of many diseases, including type 2 diabetes mellitus, NAFLD, hypertension, myocardial infarction, stroke, osteoarthritis, and cancer (82). Recently, scRNA-seq analysis of the immune cells in murine lipid-associated macrophages (LAMs) (83), NASH-associated macrophages (84), and aortic macrophages (85) indicated that a subset of macrophages expressing triggering receptor expressed on myeloid cells 2 (TREM2), which drives a gene expression program involved in phagocytosis, lipid catabolism, and energy metabolism. Further study showed that TREM2^+^ macrophages arose from circulating monocytes and positioned around enlarged adipocytes and scRNA-seq data of human adipose tissue indicated that LAM cells, as well as the TREM2 pathway, were highly conserved (83). TREM2 deletion abrogated macrophage recruitment to enlarged adipocytes and caused massive adipocyte hypertrophy, systemic hypercholesterolemia, inflammation, and glucose intolerance, indicating the protective role of TREM2^+^ macrophages (83). TREM2^−/−^ mice fed a high-fat diet (HFD) exhibited a reduction of infiltrating F4/80^+^CD11c^+^ macrophages in adipose tissue but displayed augmentation of pro-inflammatory cytokines IL-1β, IL-6, and inducible nitric oxide synthase (iNOS), adipocyte hypertrophy, hepatic steatosis, and insulin resistance compared with WT controls (86).

TREM2 expression promotes the transition of liver macrophages from the pro-inflammatory to the tissue repair phase and impacts endothelial cell differentiation during tissue recovery in mouse models of acetaminophen (acute) or CCl_4_ (chronic)-induced hepatotoxic injury (87). In addition, TREM2 expression prevents lung macrophage apoptosis during acute parainfluenza virus infections. A more active soluble form of TREM2 (sTREM2) was found after clearance of infection, which is unexpectedly active in preventing macrophage apoptosis (88).

However, the function of TREM2 may change according to disease stage and location, with several studies suggesting CNS-specific functions of TREM2. Recent genetic studies show that TREM2 mutation is also associated with a higher risk of Alzheimer's disease (AD) and multiple neurodegenerative disorders (89). In mice, TREM2 deficiency enhanced macrophage activation near the lesion of traumatic brain injury (TBI) but significantly reduced macrophage activation distant from the lesion compared to control groups in the acute stage (90). In addition, TREM2 deficiency resulted in a protective function at later time points, as the TREM2^−/−^ mice showed a reduction of hippocampal atrophy and rescue of TBI-induced behavioral changes compared to wild-type mice.

## Others

In addition to the molecules as described above, there are several less well-studied molecules expression in macrophages, including osteopontin (encoded by gene Spp1) (12), CLEC2 (C-type lectin domain family 1 member B, encoded by gene Clec1b) (91), and CD48 (92). Overall, understanding the molecular mechanism of macrophages in tissue microenvironment is critical to design macrophage-mediated therapy in diseases.

## Prognostic Markers

Two of the best studies on macrophage-associated prognostic markers include soluble CD163 (sCD163)^+^ and CD204^+^ TAMs, with particular utility in the context of liver disease and various cancers, respectively. Soluble CD163 (sCD163) is upregulated during macrophage proliferation and activation and is a marker for diagnosis of the severity and progression of liver disease. For instance, Lidofsky et al. (65) reported that serum sCD163 from macrophages in patients infected with human immunodeficiency virus (HIV) and hepatitis C virus (HCV) was positively associated with the severity of liver fibrosis from mild to moderate stage, with an Ishak fibrosis score up to 4, but not in established cirrhosis. These results suggest that sCD163 is a dynamic biomarker of hepatic fibrogenesis rather than cirrhosis in patients with viral infections (65). Another study found that sCD163 was associated with primary biliary cholangitis disease severity and long-term (a median of 8.6 years) prognosis, following a study in 201 patients (66). Besides, using sCD163 as a marker increased the prediction accuracy of poor outcomes of PBC. The sCD163 levels were also elevated in patients with early allograft dysfunction after liver transplantation, as macrophages were activated in the implanted liver when exposed to ischemia and reperfusion injury (67).

Accumulating evidence reveals that CD204^+^ TAMs promote cancer cell proliferation, invasion, and metastasis, resulting in a poor survival rate. Kawajiri and colleagues reported that a high number of CD204^+^ TAMs was associated with poorer 3-year overall survival (OS) and cumulative incidences of relapse, and a poorer prognosis in allogeneic hematopoietic cell transplantation for malignant lymphomas, such as T-cell lymphoma and leukemia (68). In lung adenocarcinoma, the expression of CD204 in TAMs was associated with a low 5-year disease-free survival rate and the aggressiveness of lung adenocarcinoma (69). CD204^+^ TAMs located in the tumor stroma area were also identified as useful prognostic markers in non-small-cell lung cancer (NSCLC) (70) and an increased number of CD204^+^ TAMs was positively associated with worse clinical prognoses in breast cancer, including relapse-free survival, distant relapse-free survival and breast cancer-specific survival (71). CD204 is also a useful marker for TAMs contributing to the angiogenesis, progression and prognosis of esophageal squamous cell carcinomas (72).

Dual markers CD163 and CD204 can also be used in diagnosis (Table 2). For instance, the numbers and percentages of M2-polarized alveolar macrophages expressing markers CD163^+^, CD204^+^, and CD206^+^ increases with the severity of chronic obstructive pulmonary disease, with higher numbers in smoker patients than non-smokers (73). In clinical findings, the number of CD163^+^CD204^+^ TAMs is negatively correlated with that of CD25^+^ cells (presumably activated lymphocytes) and 5-year progression-free survival. Dual CD163^+^CD204^+^ TAMs possibly play a vital role in the invasion and metastasis of oral squamous cell carcinoma by T-cell regulation via IL-10 and PD-L1 production, relative to CD163^+^CD204^−^ TAMs or CD163^−^CD204^+^ TAMs (74).

**Table 2:** Macrophage expressing markers CD163, CD204, or both in disease diagnosis.

**Markers**	**Diseases**	**Function**	**References**
CD163	Liver fibrosis	Under the coinfection with human immunodeficiency virus (HIV) or hepatitis C virus (HCV), serum (s)CD163 levels accompanied periportal CD163+ macrophage enrichment was associated with mild to moderate fibrosis, but not cirrhosis.	(65)
	Primary biliary cholangitis (PBC)	sCD163 represents a non-invasive measure of that provides useful long-term prognostic for PBC.	(66)
	Early allograft dysfunction (EAD)	The sCD163 levels in patients were also elevated in patients with early allograft dysfunction (EAD) after liver transplantation, as macrophages were activated in the implanted liver when exposed to ischemia and reperfusion injury.	(67)
CD204	Malignant lymphomas, such as T-cell lymphoma and leukemia	A high number of CD204^+^ TAMs was associated with 3-year poorer overall survival (OS) and cumulative incidences of relapse, and the poorer prognosis in allogeneic hematopoietic cell transplantation for malignant lymphomas.	(68)
	Lung adenocarcinoma	The expression of CD204 in TAMs was associated with a low 5-year disease-free survival (DFS) rate and the aggressiveness of lung adenocarcinoma. In lung adenocarcinoma, CD204^+^ TAMs located in the tumor stroma area were the preferable marker for prognostic prediction in non-small-cell lung cancer (NSCLC).	(69, 70)
	Breast cancer (BC)	An increased number of CD204^+^ TAMs was positively associated with worse clinical prognoses in breast cancer, including relapse-free survival, distant relapse-free survival and breast cancer-specific survival.	(71)
	Esophageal squamous cell carcinomas (ESCCs)	CD204 is a useful marker for TAMs contributing to the angiogenesis, progression and prognosis of esophageal squamous cell carcinomas (ESCCs).	(72)
CD163 and CD204	Chronic obstructive pulmonary disease (COPD)	The numbers and percentages of M2 markers CD163^+^CD204^+^ alveolar macrophages were positively associated with the severity of chronic obstructive pulmonary disease (COPD), with higher numbers in smoker patients than non-smokers.	(73)
	Oral squamous cell carcinoma (OSCC)	Dual CD163^+^CD204^+^ TAMs possibly play a key role in the invasion and metastasis of oral squamous cell carcinoma (OSCC) by T-cell regulation via IL-10 and PD-L1 production, more valuable than CD163^+^CD204^−^ TAMs or CD163^−^CD204^+^ TAMs.	(74)

## Therapeutic Targets for Diseases

While M2-like pro-tumoral phenotype is dominant in tumor microenvironment, targeting TAMs or conversion of TAMs to an M1-like anti-tumoral phenotype is an emerging strategy for targeting TAMs-mediated cancer therapy (93). An *in vivo* assay showed that a hybrid peptide composed of melittin and the pro-apoptotic peptide, selectively targeted TAMs without impacting affecting other leukocytes, such as T cells and dendritic cells. Treatment of the hybrid peptide resulted in apoptosis of CD206^+^ M2-like TAMs and reduction of tumor growth and angiogenesis, showing as a promising cancer therapeutic agent (94).

**Figure 2:**
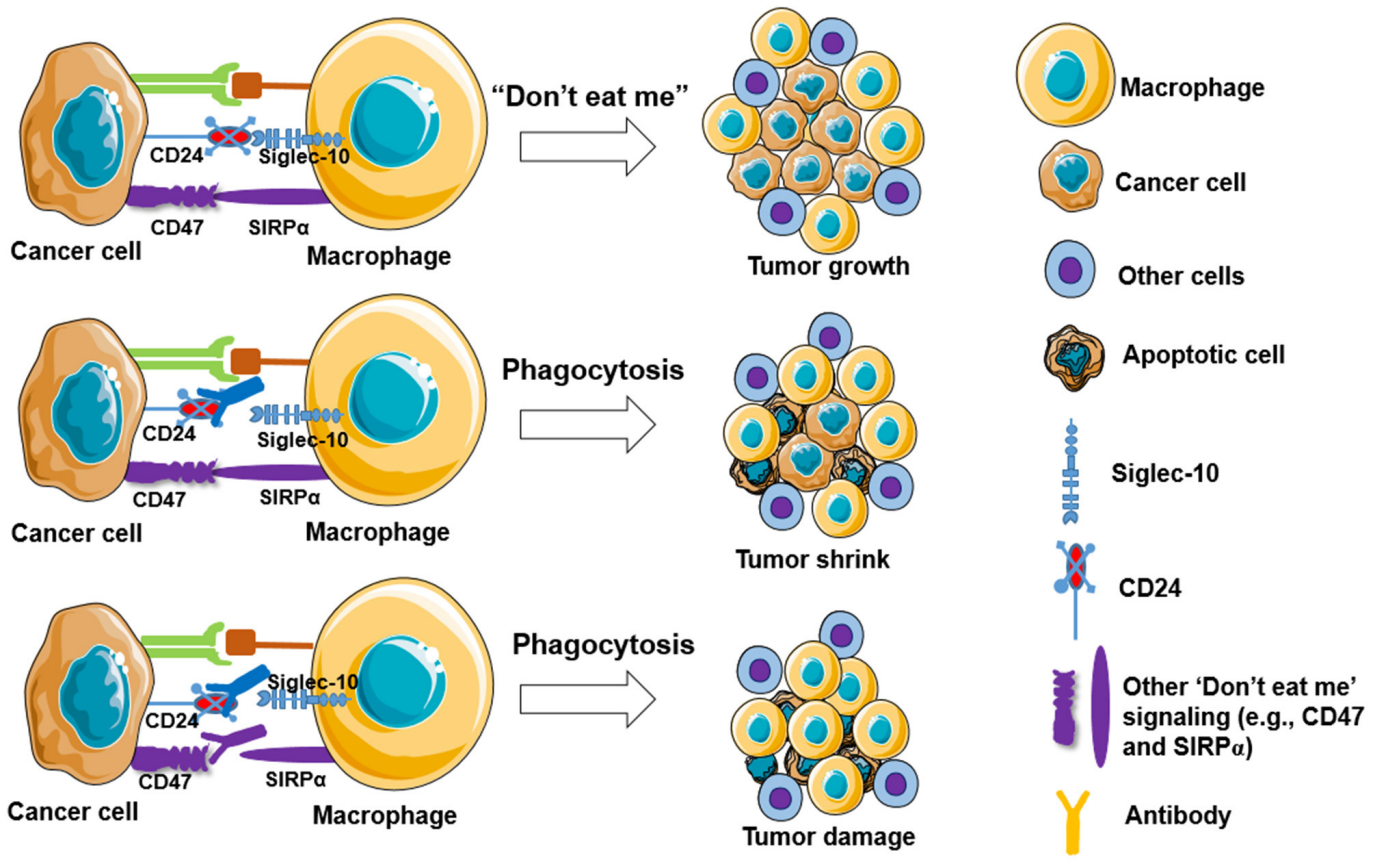
Blockade of “Don't eat me” signalings increases macrophage phagocytosis to tumor cells. Cancer cells are capable of evading macrophage phagocytosis by expressing “don't eat me” signals, including CD47, PD-L1 (CD274), the beta-microglobulin subunit of the major histocompatibility class I complex (B2M), and CD24, resulting in tumor growth (top panel). Partly blockade (middle panel) and fully blockade (low panel) of these “Don't eat me” signalings may induce tumor shrink and tumor damage, respectively.

Targeting “Don't eat me” signaling pathway such as blocking the CD47-SIRPα interaction can improve macrophage phagocytosis of tumor cells. Monotherapy of TTI-621, a fusion protein consisting of CD47 binding domain of SIRPα, is well-tolerated and shows rapidly active responses in adult patients with relapsed/refractory percutaneously accessible solid tumors (95). Another study showed that anti-human SIRPα monoclonal antibody (Ab) KWAR23 treatment alone does not increase human macrophage phagocytosis of CD20-expressing lymphoma cells, but significantly augments phagocytosis of lymphoma cells when combined with treatment of anti-CD20 antibodies (e.g., rituximab) *in vitro*, and this effect was also shown in a lymphoma xenograft mouse model expressing human SIRPα (96). Murata et al. (97) recently showed that anti-human SIRPα Ab significantly enhances macrophage phagocytosis of lymphoma cancer cells in immunodeficient mice harboring a transgene of human SIRPα, which also shows a synergistic effect of rituximab on inhibition of tumor growth. In addition, anti-mouse SIRPα (P84 or MY-1) Ab treatment markedly inhibits the growth of tumors formed by SIRPα-expressing renal cell carcinoma and melanoma in mice. Moreover, the anti-SIRPα Ab treatment shows a synergistic effect in combination with rituximab and anti-programmed cell death 1 (PD-1) on tumor formation in mice (98). Other strategies have been applied to improve macrophage-mediated anti-tumor responses and reduce the toxicity to normal cells, such as using exosomes harboring SIRPα to block the interaction between CD47 and SIRPα (99) and augmenting the contact interface of CD47-SIRPα via engineering N-terminal peptide extension (80). In addition, new signaling pathways are also reported to function similarly. For example, Barkal et al. (61) reported that CD24 signaling through macrophage Siglec-10 is a potential therapeutic target for cancer immunotherapy (Figure 2), as blockade of CD24-Siglec-10 signaling can promote macrophage phagocytosis of triple-negative breast cancer and ovarian cancer cells (61).

Cai et al. (100) reported that MerKT in liver macrophage contributes to liver fibrosis since MerKT-deficient mice slowed the development of liver fibrosis compared to wild-type mice when fed a NASH-promoting diet. Molecular study showed an increase of MerKT expression on macrophages induced TGF-b1 expression and secretion via ERK1/2 signaling, which promoted hepatic stellate cell activation to produce collagen production. The authors also found that blockade of the interaction of MerKT with its ligand Gas6 using RU-30, an inhibitor of receptors (Tyro-3, Axl, and Mertk), significantly inhibited the production of collagen.

## Strategies to Study the Function of Macrophages

To study the precise functions of macrophages under different physiological and pathological conditions, several strategies have been used to deplete macrophages in various mouse models, including genetic ablation (101), nanotechnology-based depletion by clodronate-encapsulated liposomes (102), and antibody-mediated depletion (103). These strategies have produced encouraging results in experimental murine models. For example, in transgenic mice based on CD11b promoter-mediated expression of the human diphtheria toxin (DT) receptor, transient depletion of macrophages occurs *in vivo* when DT is injected. Using this model, the researchers identified that functionally distinct subpopulations of macrophages exist in liver injury and recovery, inducing liver fibrosis by promoting extracellular matrix deposition during liver injury or promoting the resolution of liver fibrosis during recovery (104). These methods are however not perfectly specific for macrophages and induce only temporary ablation.

Clodronate-encapsulated liposomes have been broadly applied to deplete macrophages in different tissues (bone marrow, spleen, liver, lungs, brain, gut, peritoneal cavity, lymph nodes/vessels) and blood. A protocol used to deplete macrophages in mice was described before (102). For instance, a single i.p. injection of clodronate liposome during chronic cholangiopathy resulted in significant inhibition of ECM deposition in mice (105). In a murine model of Azoxymethane (AOM)/Dextran Sodium Sulfate (DSS)-induced colorectal cancer, mice received multiple treatments with clodronate to deplete macrophages resulted in a significant decrease of tumor number by ~35%, specifically for large (≥1 mm) tumors. This effect was accompanied with a decrease in gene expression of pan macrophage marker F4/80 as well as expression of markers associated with M2 macrophages (IL-13, IL-10, TGF-β, and CCL17) (106). In addition, macrophage depletion was also associated with a significantly increased relative abundance of the *Firmicutes* phylum in stool, demonstrating that macrophages are important mediators of tumor growth and directly or indirectly capable of influencing the gut microbiota during colorectal cancer development (106). TAMs increased as urethane-induced lung tumor grew in mice, which exhibited a mixed M1/M2-like macrophage phenotype. Liposomal clodronate treatment (one time of intrathecal administration plus five times of i.v. administration) significantly decreased the alveolar macrophage population (> 50%) and resulted in a 50% reduction in tumor burden compared to vehicle liposome-treated mice (107). A subsequent study demonstrated that inhibition of recruited macrophages by blockade of CCL2/CCR2 signaling did not attenuate lung cancer progression, as CCR2-deficient mice showed similar tumor growth rate compared to wild-type mice.

However, depletion of the entire macrophages in a specific tissue or whole body may result in unwanted side effects. For instance, depletion of peritoneal macrophages with clodronate injection induced a dramatic decrease in neutrophil recruitment in experimental peritonitis (108). Another study showed transient depletion of macrophages may exacerbate liver injury in NASH patients since monocytes-derived KCs show more pro-inflammatory activity and are less efficient in hepatic triglyceride storage (91). As we discussed above molecular markers, some subpopulations of macrophages also benefit the resolution of diseases, such as LYVE-1+ macrophages. Thus, deletion of specific macrophage subpopulation or specific gene in macrophages under investigation may represent a more reasonable approach.

## Summary

Macrophages are a key population of innate immunity, with powerful influences on homeostasis, tissue repair, obesity, and cancer. Macrophages consist of two populations, tissue-resident macrophages with a prenatal origin and postnatal monocyte-derived macrophages. Independent of their origin, macrophages are very plastic cells, being able to change their phenotype according to local environmental stimuli. They are usually polarized into M1-like or M2-like phenotype; however, recent studies suggest that the phenotype of macrophages cannot be simply divided into M1/M2 dichotomy, and additional classifications, such as TAMs and ATMs, are applied to define the tissue-specific macrophages. In this review, promising candidate macrophage markers are highlighted due to their potential application in diagnosis and treatment against diseases.

Several methods have been applied to target TAMs or ATMs to prevent tumor growth or inflammation, such as macrophage depletion, blockade of anti-phagocytic signaling (e.g., Siglec-10 or SIRPα). Therefore, understanding the molecular mechanisms of macrophage function in disease provides potent diagnostic markers and/or therapeutic strategies.

## Author Contributions

CZ and MY conceived and wrote the manuscript. AE provided guidance for part of manuscript, critically reviewed and significantly edited the manuscript. All authors contributed to the article and approved the submitted version.

## Conflict of Interest

The authors declare that the research was conducted in the absence of any commercial or financial relationships that could be construed as a potential conflict of interest.

## References

[1] Epelman S Lavine KJ Randolph GJ. Origin and functions of tissue macrophages. Immunity. (2014) 41:21–35. 10.1016/j.immuni.2014.06.013 25035951 PMC4470379

[2] Yona S Kim KW Wolf Y Mildner A Varol D Breker M. Fate mapping reveals origins and dynamics of monocytes and tissue macrophages under homeostasis. Immunity. (2013) 38:79–91. 10.1016/j.immuni.2012.12.001 23273845 PMC3908543

[3] Hashimoto D Chow A Noizat C Teo P Beasley MB Leboeuf M. Tissue-resident macrophages self-maintain locally throughout adult life with minimal contribution from circulating monocytes. Immunity. (2013) 38:792–804. 10.1016/j.immuni.2013.04.004 23601688 PMC3853406

[4] Gomez Perdiguero Klapproth EK Schulz C Busch K Azzoni E Crozet L Garner HC. Tissue-resident macrophages originate from yolk-sac-derived erythro-myeloid progenitors. Nature. (2015) 518:547–51. 10.1038/nature13989 25470051 PMC5997177

[5] Epelman S Lavine KJ Beaudin AE Sojka DK Carrero JA Calderon B. Embryonic and adult-derived resident cardiac macrophages are maintained through distinct mechanisms at steady state and during inflammation. Immunity. (2014) 40:91–104. 10.1016/j.immuni.2013.11.019 24439267 PMC3923301

[6] Wang X Sathe AA Smith GR Ruf-Zamojski F Nair V Lavine KJ. Heterogeneous origins and functions of mouse skeletal muscle-resident macrophages. Proc Natl Acad Sci U S A. (2020) 117:20729–40. 10.1073/pnas.1915950117 32796104 PMC7456122

[7] Saeki N Imai Y. Reprogramming of synovial macrophage metabolism by synovial fibroblasts under inflammatory conditions. Cell Commun Signal. (2020) 18:188. 10.1186/s12964-020-00678-8 33256735 PMC7708128

[8] Herzog C Pons Garcia L Keatinge M Greenald D Moritz C Peri F. Rapid clearance of cellular debris by microglia limits secondary neuronal cell death after brain injury in vivo. Development. 146:9. (2019) 10.1242/dev.174698 31076485 PMC6526721

[9] Bosurgi L Cao YG Cabeza-Cabrerizo M Tucci A Hughes LD Kong Y. Macrophage function in tissue repair and remodeling requires IL-4 or IL-13 with apoptotic cells. Science. (2017) 356:1072–6. 10.1126/science.aai8132 28495875 PMC5556699

[10] Greenhalgh AD Zarruk JG Healy LM Baskar Jesudasan SJ Jhelum P Salmon CK. Peripherally derived macrophages modulate microglial function to reduce inflammation after CNS injury. PLoS Biol. (2018) 16:e2005264. 10.1371/journal.pbio.2005264 30332405 PMC6205650

[11] Fastrès A Pirottin D Fievez L Tutunaru A-C Bolen G Merveille A-C. Identification of pro-fibrotic macrophage populations by single-cell transcriptomic analysis in west highland white terriers affected with canine idiopathic pulmonary fibrosis. Front Immunol. (2020) 11:611749. 10.3389/fimmu.2020.611749 33384697 PMC7770158

[12] Remmerie A Martens L Thoné T Castoldi A Seurinck R Pavie B. Osteopontin expression identifies a subset of recruited macrophages distinct from Kupffer cells in the fatty liver. Immunity. (2020) 53:641–57. 10.1016/j.immuni.2020.08.004 32888418 PMC7501731

[13] Sárvári AK Van Hauwaert EL Markussen LK Gammelmark E Marcher AB Ebbesen MF. Plasticity of epididymal adipose tissue in response to diet-induced obesity at single-nucleus resolution. Cell Metab. (2020) 10.1016/j.cmet.2020.12.004 33378646

[14] Liu D Guo M Zhou P Xiao J Ji X. TSLP promote M2 macrophages polarization and cardiac healing after myocardial infarction. Biochem Biophys Res Commun. (2019) 516:437–44. 10.1016/j.bbrc.2019.06.041 31227217

[15] Van Hove Martens L Scheyltjens I De Vlaminck K Pombo Antunes AR De Prijck S Vandamme N. A single-cell atlas of mouse brain macrophages reveals unique transcriptional identities shaped by ontogeny and tissue environment. Nat Neurosci. (2019) 22:1021–35. 10.1038/s41593-019-0393-4 31061494

[16] Mills CD Kincaid K Alt JM Heilman MJ Hill AM. M-1/M-2 macrophages and the Th1/Th2 paradigm. J Immunol. (2000) 164:6166–73. 10.4049/jimmunol.164.12.6166 10843666

[17] Chen Y Zhang X. Pivotal regulators of tissue homeostasis and cancer: macrophages. Exp Hematol Oncol. (2017) 6:23. 10.1186/s40164-017-0083-4 28804688 PMC5549331

[18] Gordon S Martinez FO. Alternative activation of macrophages: mechanism and functions. Immunity. (2010) 32:593–604. 10.1016/j.immuni.2010.05.007 20510870

[19] Novak ML Koh TJ. Macrophage phenotypes during tissue repair. J Leukocyte Biol. (2013) 93:875–81. 10.1189/jlb.1012512 23505314 PMC3656331

[20] Orecchioni M Ghosheh Y Pramod AB Ley K. Macrophage polarization: different gene signatures in M1(LPS+) vs. classically and M2(LPS-) vs. alternatively activated macrophages. Front Immunol. (2019) 10:1084. 10.3389/fimmu.2019.01084 31178859 PMC6543837

[21] Trombetta AC Soldano S Contini P Tomatis V Ruaro B Paolino S. A circulating cell population showing both M1 and M2 monocyte/macrophage surface markers characterizes systemic sclerosis patients with lung involvement. Resp Res. (2018) 19:186. 10.1186/s12931-018-0891-z 30249259 PMC6154930

[22] Liu SX Gustafson HH Jackson DL Pun SH Trapnell C. Trajectory analysis quantifies transcriptional plasticity during macrophage polarization. Sci Rep. (2020) 10:12273. 10.1038/s41598-020-68766-w 32703960 PMC7378057

[23] Viola A Munari F Sánchez-Rodríguez R Scolaro T Castegna A. The metabolic signature of macrophage responses. Front Immunol. (2019) 10:1462. 10.3389/fimmu.2019.01462 31333642 PMC6618143

[24] Jha AK Huang SC Sergushichev A Lampropoulou V Ivanova Y Loginicheva E. Network integration of parallel metabolic and transcriptional data reveals metabolic modules that regulate macrophage polarization. Immunity. (2015) 42:419–30. 10.1016/j.immuni.2015.02.005 25786174

[25] Hill DA Lim H-W Kim YH Ho WY Foong YH Nelson VL. Distinct macrophage populations direct inflammatory versus physiological changes in adipose tissue. Proc Natl Acad Sci USA. (2018) 115:E5096. 10.1073/pnas.1802611115 29760084 PMC5984532

[26] Kratz M Coats BR Hisert KB Hagman D Mutskov V Peris E. Metabolic dysfunction drives a mechanistically distinct proinflammatory phenotype in adipose tissue macrophages. Cell Metab. (2014) 20:614–25. 10.1016/j.cmet.2014.08.010 25242226 PMC4192131

[27] Wenes M Shang M Di Matteo M Goveia J Martín-Pérez R Serneels J. Macrophage metabolism controls tumor blood vessel morphogenesis and metastasis. Cell Metab. (2016) 24:701–15. 10.1016/j.cmet.2016.09.008 27773694

[28] Tu D Dou J Wang M Zhuang H Zhang X. M2 macrophages contribute to cell proliferation and migration of breast cancer. Cell Biol Int. (2020). 10.21203/rs.3.rs-39373/v1. [Epub ahead of print]. 33325089

[29] Ruffell B Chang-Strachan D Chan V Rosenbusch A Ho CM Pryer N. Macrophage IL-10 blocks CD8+ T cell-dependent responses to chemotherapy by suppressing IL-12 expression in intratumoral dendritic cells. Cancer Cell. (2014) 26:623–37. 10.1016/j.ccell.2014.09.006 25446896 PMC4254570

[30] Yuan H Lin Z Liu Y Jiang Y Liu K Tu M. Intrahepatic cholangiocarcinoma induced M2-polarized tumor-associated macrophages facilitate tumor growth and invasiveness. Cancer Cell Int. (2020) 20:586. 10.1186/s12935-020-01687-w 33372604 PMC7720384

[31] Wang XL Jiang JT Wu CP. Prognostic significance of tumor-associated macrophage infiltration in gastric cancer: a meta-analysis. Genet Mol Res. 15. (2016) 10.4238/gmr15049040 27966749

[32] Landry AP Balas M Alli S Spears J Zador Z. Distinct regional ontogeny and activation of tumor associated macrophages in human glioblastoma. Sci Rep. (2020) 10:19542. 10.1038/s41598-020-76657-3 33177572 PMC7658345

[33] Mould KJ Jackson ND Henson PM Seibold M Janssen WJ. Single cell RNA sequencing identifies unique inflammatory airspace macrophage subsets. JCI Insight. 4:5. (2019) 10.1172/jci.insight.126556 30721157 PMC6483508

[34] Etzerodt A Moestrup SK. CD163 and inflammation: biological, diagnostic, therapeutic aspects. Antioxid Redox Sign. (2013) 18:2352–63. 10.1089/ars.2012.4834 22900885 PMC3638564

[35] Buechler C Ritter M Orsó E Langmann T Klucken J Schmitz G. Regulation of scavenger receptor CD163 expression in human monocytes and macrophages by pro- and antiinflammatory stimuli. J Leukoc Biol. (2000) 67:97–103. 10.1002/jlb.67.1.97 10648003

[36] Shiraishi D Fujiwara Y Horlad H Saito Y Iriki T Tsuboki J. CD163 is required for protumoral activation of macrophages in human and murine sarcoma. Cancer Res. (2018) 78:3255–66. 10.1158/0008-5472.CAN-17-2011 29610117

[37] Ramos RN Rodriguez C Hubert M Ardin M Treilleux I Ries CH. CD163(+) tumor-associated macrophage accumulation in breast cancer patients reflects both local differentiation signals and systemic skewing of monocytes. Clin Transl Immunol. (2020) 9:e1108. 10.1002/cti2.1108 32082570 PMC7017151

[38] Pinto ML Rios E Durães C Ribeiro R Machado JC Mantovani A. The two faces of tumor-associated macrophages and their clinical significance in colorectal cancer. Front Immunol. (2019) 10:1875. 10.3389/fimmu.2019.01875 31481956 PMC6710360

[39] Rozek LS Schmit SL Greenson JK Tomsho LP Rennert HS Rennert G. Tumor-Infiltrating lymphocytes, Crohn's-like lymphoid reaction, and survival from colorectal cancer. J Natl Cancer Inst. (2016) 108:27. 10.1093/jnci/djw027 27172903 PMC5017930

[40] Banerji S Ni J Wang SX Clasper S Su J Tammi R. LYVE-1, a new homologue of the CD44 glycoprotein, is a lymph-specific receptor for hyaluronan. J Cell Biol. (1999) 144:789–801. 10.1083/jcb.144.4.789 10037799 PMC2132933

[41] Lim HY Lim SY Tan CK Thiam CH Goh CC Carbajo D. Hyaluronan receptor LYVE-1-expressing macrophages maintain arterial tone through hyaluronan-mediated regulation of smooth muscle cell collagen. Immunity. (2018) 49:326–41.e7. 10.1016/j.immuni.2018.06.008 30054204

[42] Brezovakova V Jadhav S. Identification of Lyve-1 positive macrophages as resident cells in meninges of rats. J Comp Neurol. (2020) 528:2021–32. 10.1002/cne.24870 32003471

[43] Dollt C Becker K Michel J Melchers S Weis C-A Schledzewski K. The shedded ectodomain of Lyve-1 expressed on M2-like tumor-associated macrophages inhibits melanoma cell proliferation. Oncotarget. (2017) 8:103682. 10.18632/oncotarget.21771 29262593 PMC5732759

[44] Oe S Masum MA Ichii O Nishimura T Nakamura T Namba T. Spatiotemporal histological changes observed in mouse subcutaneous tissues during the foreign body reaction to silicone. J Biomed Mater Res A. (2020). 10.1002/jbm.a.37115. [Epub ahead of print]. 33021053

[45] Xu H Chen M Reid DM Forrester JV. LYVE-1-positive macrophages are present in normal murine eyes. Invest Ophthalmol Vis Sci. (2007) 48:2162–71. 10.1167/iovs.06-0783 17460275

[46] Kurowska-Stolarska M Alivernini S. Synovial tissue macrophages: friend or foe? RMD Open. (2017) 3:e000527. 10.1136/rmdopen-2017-000527 29299338 PMC5729306

[47] Alivernini S MacDonald L Elmesmari A Finlay S Tolusso B Gigante MR. Distinct synovial tissue macrophage subsets regulate inflammation and remission in rheumatoid arthritis. Nature Medicine. (2020) 26:1295–306. 10.1038/s41591-020-0939-8 32601335

[48] Kuo D Ding J Cohn IS Zhang F Wei K Rao DA. HBEGF(+) macrophages in rheumatoid arthritis induce fibroblast invasiveness. Sci Transl Med. (2019) 11:eaau8587. 10.1126/scitranslmed.aau8587 31068444 PMC6726376

[49] Lantz C Radmanesh B Liu E Thorp EB Lin J. Single-cell RNA sequencing uncovers heterogenous transcriptional signatures in macrophages during efferocytosis. Scientific Reports. (2020) 10:14333. 10.1038/s41598-020-70353-y 32868786 PMC7459098

[50] von Gunten S Bochner BS. Basic and clinical immunology of Siglecs. Ann N Y Acad Sci. (2008) 1143:61–82. 10.1196/annals.1443.011 19076345 PMC3902170

[51] Clancy RM Halushka M Rasmussen SE Lhakhang T Chang M Buyon JP. Siglec-1 macrophages and the contribution of IFN to the development of autoimmune congenital heart block. J Immunol. (2019) 202:48–55. 10.4049/jimmunol.1800357 30518570 PMC6310077

[52] Pluvinage JV Haney MS Smith BAH Sun J Iram T Bonanno L. CD22 blockade restores homeostatic microglial phagocytosis in ageing brains. Nature. (2019) 568:187–192. 10.1038/s41586-019-1088-4 30944478 PMC6574119

[53] Bhattacherjee A Rodrigues E Jung J Luzentales-Simpson M Enterina JR Galleguillos DCD. Repression of phagocytosis by human CD33 is not conserved with mouse CD33. Commun Biol. (2019) 2:450. 10.1038/s42003-019-0698-6 31815204 PMC6890642

[54] Pan B Fromholt SE Hess EJ Crawford TO Griffin JW Sheikh KA. Myelin-associated glycoprotein and complementary axonal ligands, gangliosides, mediate axon stability in the CNS and PNS: neuropathology and behavioral deficits in single- and double-null mice. Exp Neurol. (2005) 195:208–217. 10.1016/j.expneurol.2005.04.017 15953602 PMC1852502

[55] Wielgat P Mroz RM Stasiak-Barmuta A Szepiel P Chyczewska E Braszko JJ. Inhaled corticosteroids increase siglec-5/14 expression in sputum cells of COPD patients. Adv Exp Med Biol. (2015) 839:1–5. 10.1007/5584_2014_51 25252903

[56] Tsai CM Riestra AM Ali SR Fong JJ Liu JZ Hughes G. Siglec-14 enhances NLRP3-inflammasome activation in macrophages. J Innate Immun. (2019) 2019:1-11. 10.1159/000504323 31805552 PMC7383293

[57] Yu Y Blokhuis BRJ Diks MAP Keshavarzian A Garssen J Redegeld FA. Functional inhibitory siglec-6 is upregulated in human colorectal cancer-associated mast cells. Front Immunol. (2018) 9:2138. 10.3389/fimmu.2018.02138 30294327 PMC6159741

[58] Sakamoto Y Yoshio S Doi H Kawai H Shimagaki T Mori T. Serum soluble sialic acid-binding immunoglobulin-like lectin-7 concentration as an indicator of liver macrophage activation and advanced fibrosis in patients with non-alcoholic fatty liver disease. Hepatol Res. (2020) 50:466–77. 10.1111/hepr.13464 31808236

[59] Johansson MW Kelly EA Nguyen CL Jarjour NN Bochner BS. Characterization of Siglec-8 expression on lavage cells after segmental lung allergen challenge. Int Arch Allergy Immunol. (2018) 177:16–8. 10.1159/000488951 29879704 PMC6105496

[60] Chu S Zhu X You N Zhang W Zheng F Cai B. The fab fragment of a human anti-siglec-9 monoclonal antibody suppresses LPS-induced inflammatory responses in human macrophages. Front Immunol. (2016) 7:649. 10.3389/fimmu.2016.00649 28082984 PMC5183739

[61] Barkal AA Brewer RE Markovic M Kowarsky M Barkal SA Zaro BW. CD24 signalling through macrophage Siglec-10 is a target for cancer immunotherapy. Nature. (2019) 572:392–6. 10.1038/s41586-019-1456-0 31367043 PMC6697206

[62] Shahraz A Kopatz J Mathy R Kappler J Winter D Kapoor S. Anti-inflammatory activity of low molecular weight polysialic acid on human macrophages. Sci Rep. (2015) 5:16800. 10.1038/srep16800 26582367 PMC4652165

[63] Mitra N Banda K Altheide TK Schaffer L Johnson-Pais TL Beuten J. SIGLEC12, a human-specific segregating (pseudo)gene, encodes a signaling molecule expressed in prostate carcinomas. J Biol Chem. (2011) 286:23003–11. 10.1074/jbc.M111.244152 21555517 PMC3123068

[64] Takamiya R Ohtsubo K Takamatsu S Taniguchi N Angata T. The interaction between Siglec-15 and tumor-associated sialyl-Tn antigen enhances TGF-β secretion from monocytes/macrophages through the DAP12-Syk pathway. Glycobiology. (2013) 23:178–87. 10.1093/glycob/cws139 23035012

[65] Lidofsky A Holmes JA Feeney ER Kruger AJ Salloum S Zheng H. Macrophage activation marker soluble CD163 is a dynamic marker of liver fibrogenesis in human immunodeficiency virus/hepatitis C virus coinfection. J Infect Dis. (2018) 218:1394–403. 10.1093/infdis/jiy331 29868909 PMC6151081

[66] Bossen L Rebora P Bernuzzi F Jepsen P Gerussi A Andreone P. Soluble CD163 and mannose receptor as markers of liver disease severity and prognosis in patients with primary biliary cholangitis. Liver Int. (2020) 40:1408–14. 10.1111/liv.14466 32279422

[67] Thomsen KL Robertson FP Holland-Fischer P Davidson BR Mookerjee RP Moller HJ. The macrophage activation marker soluble CD163 is associated with early allograft dysfunction after liver transplantation. J Clin Exp Hepatol. (2019) 9:302–11. 10.1016/j.jceh.2018.09.006 31360022 PMC6637071

[68] Kawajiri A Kitano S Maeshima AM Inamoto Y Tajima K Takemura T. Association of CD204(+) macrophages with poor outcomes of malignant lymphomas not in remission treated by allogeneic HCT. Eur J Haematol. (2019) 103:578–87. 10.1111/ejh.13324 31487403

[69] Sun Y Xu S. Tumor-Associated CD204-positive macrophage is a prognostic marker in clinical stage i lung adenocarcinoma. Biomed Res Int. (2018) 2018:8459193. 10.1155/2018/8459193 29850577 PMC5926519

[70] Li Z Maeda D Yoshida M Umakoshi M Nanjo H Shiraishi K. The intratumoral distribution influences the prognostic impact of CD68- and CD204-positive macrophages in non-small cell lung cancer. Lung Cancer. (2018) 123:127–135. 10.1016/j.lungcan.2018.07.015 30089583

[71] Miyasato Y Shiota T Ohnishi K Pan C Yano H Horlad H. High density of CD204-positive macrophages predicts worse clinical prognosis in patients with breast cancer. Cancer science. (2017) 108:1693–700. 10.1111/cas.13287 28574667 PMC5543503

[72] Shigeoka M Urakawa N Nakamura T Nishio M Watajima T Kuroda D. Tumor associated macrophage expressing CD204 is associated with tumor aggressiveness of esophageal squamous cell carcinoma. Cancer Science. (2013) 104:1112–9. 10.1111/cas.12188 23648122 PMC7657117

[73] Kaku Y Imaoka H Morimatsu Y Komohara Y Ohnishi K Oda H. Overexpression of CD163, CD204 and CD206 on alveolar macrophages in the lungs of patients with severe chronic obstructive pulmonary disease. PLoS ONE. (2014) 9:e87400. 10.1371/journal.pone.0087400 24498098 PMC3907529

[74] Kubota K Moriyama M Furukawa S Rafiul ASM H Maruse Y Jinno T. CD163+CD204+ tumor-associated macrophages contribute to T cell regulation via interleukin-10 and PD-L1 production in oral squamous cell carcinoma. Sci Rep. (2017) 7:1755. 10.1038/s41598-017-01661-z 28496107 PMC5431876

[75] Oliveira JJ Karrar S Rainbow DB Pinder CL Clarke P Rubio Garcia A. The plasma biomarker soluble SIGLEC-1 is associated with the type I interferon transcriptional signature, ethnic background and renal disease in systemic lupus erythematosus. Arthritis Res Ther. (2018) 20:152. 10.1186/s13075-018-1649-1 30053827 PMC6062988

[76] Kazankov K Jorgensen SMD Thomsen KL Moller HJ Vilstrup H George J. The role of macrophages in nonalcoholic fatty liver disease and nonalcoholic steatohepatitis. Nat Rev Gastroenterol Hepatol. (2019) 16:145–9. 10.1038/s41575-018-0082-x 30482910

[77] Ding J Zhao D Hu Y Liu M Liao X Zhao B. Terminating the renewal of tumor-associated macrophages: A sialic acid-based targeted delivery strategy for cancer immunotherapy. Int J Pharm. (2019) 571:118706. 10.1016/j.ijpharm.2019.118706 31593811

[78] Willingham SB Volkmer JP Gentles AJ Sahoo D Dalerba P Mitra SS. The CD47-signal regulatory protein alpha (SIRPa) interaction is a therapeutic target for human solid tumors. Proc Natl Acad Sci USA. (2012) 109:6662–7. 10.1073/pnas.1121623109 22451913 PMC3340046

[79] Horrigan SK. Replication study: the CD47-signal regulatory protein alpha (SIRPa) interaction is a therapeutic target for human solid tumors. Elife. (2017) 6:18173. 10.7554/eLife.18173 28100392 PMC5245970

[80] Ho CC Guo N Sockolosky JT Ring AM Weiskopf K Özkan E. Garcia: “Velcro” engineering of high affinity CD47 ectodomain as signal regulatory protein α (SIRPα) antagonists that enhance antibody-dependent cellular phagocytosis. J Biol Chem. (2015) 290:12650–63. 10.1074/jbc.M115.648220 25837251 PMC4432284

[81] Weiskopf K. Cancer immunotherapy targeting the CD47/SIRPα axis. Eur J Cancer. (2017) 76:100-9. 10.1016/j.ejca.2017.02.013 28286286

[82] Blüher M. Obesity: global epidemiology and pathogenesis. Nat Rev Endocrinol. (2019) 15:288–98. 10.1038/s41574-019-0176-8 30814686

[83] Jaitin DA Adlung L Thaiss CA Weiner A Li B Descamps H. Lipid-associated macrophages control metabolic homeostasis in a Trem2-dependent manner. Cell. (2019) 178:686–98.e14. 10.1016/j.cell.2019.05.054 31257031 PMC7068689

[84] Xiong X Kuang H Ansari S Liu T Gong J Wang S. Landscape of intercellular crosstalk in healthy and NASH liver revealed by single-cell secretome gene analysis. Mol Cell. (2019) 75:644–60.e5. 10.1016/j.molcel.2019.07.028 31398325 PMC7262680

[85] Cochain C Vafadarnejad E Arampatzi P Pelisek J Winkels H Ley K. Single-Cell RNA-Seq reveals the transcriptional landscape and heterogeneity of aortic macrophages in murine atherosclerosis. Circ Res. (2018) 122:1661–74. 10.1161/CIRCRESAHA.117.312509 29545365

[86] Liu C Li P Li H Wang S Ding L Wang H. TREM2 regulates obesity-induced insulin resistance via adipose tissue remodeling in mice of high-fat feeding. J Trans Med. (2019) 17:300. 10.1186/s12967-019-2050-9 31477129 PMC6720981

[87] Coelho I Duarte N Barros A Macedo MP Penha-Gonçalves C. Trem-2 promotes emergence of restorative macrophages and endothelial cells during recovery from hepatic tissue damage. bioRxiv. (2019) 2019:823773. 10.1101/823773 33628208 PMC7897679

[88] Wu K Byers DE Jin X Agapov E Alexander-Brett J Patel AC. TREM-2 promotes macrophage survival and lung disease after respiratory viral infection. J Exp Med. (2015) 212:681–97. 10.1084/jem.20141732 25897174 PMC4419356

[89] Gratuze M Leyns CEG Holtzman DM. New insights into the role of TREM2 in Alzheimer's disease. Mol Neurodegen. (2018) 13:66. 10.1186/s13024-018-0298-9 30572908 PMC6302500

[90] Saber M Kokiko-Cochran O Puntambekar SS Lathia JD Lamb BT. Triggering receptor expressed on myeloid cells 2 deficiency alters acute macrophage distribution and improves recovery after traumatic brain injury. J Neurotr. (2016) 34:423–35. 10.1089/neu.2016.4401 26976047

[91] Tran S Baba I Poupel L Dussaud S Moreau M Gélineau A. Impaired Kupffer cell self-renewal alters the liver response to lipid overload during non-alcoholic steatohepatitis. Immunity. (2020) 53:627–40.e5. 10.1016/j.immuni.2020.06.003 32562600

[92] Zou C Zhu C Guan G Guo Q Liu T Shen S. CD48 is a key molecule of immunomodulation affecting prognosis in glioma. Onco Targets Ther. (2019) 12:4181–93. 10.2147/OTT.S198762 31213836 PMC6549391

[93] Chen RH Xiao ZW Yan XQ Han P Liang FY Wang JY. Tumor cell-secreted ISG15 promotes tumor cell migration and immune suppression by inducing the macrophage M2-like phenotype. Front Immunol. (2020) 11:594775. 10.3389/fimmu.2020.594775 33424843 PMC7785797

[94] Lee C Jeong H Bae Y Shin K Kang S Kim H. Targeting of M2-like tumor-associated macrophages with a melittin-based pro-apoptotic peptide. J ImmunoTher Cancer. (2019) 7:147. 10.1186/s40425-019-0610-4 31174610 PMC6555931

[95] Querfeld C Thompson J Taylor MH Pillai R Johnson LD Catalano T. Intralesional administration of the CD47 antagonist TTI-621 (SIRPαFc) induces responses in both injected and non-injected lesions in patients with relapsed/refractory mycosis fungoides and Sézary syndrome: interim results of a multicenter phase i trial. Blood. (2018) 132(Supplement 1):1653. 10.1182/blood-2018-99-116915

[96] Ring NG Herndler-Brandstetter D Weiskopf K Shan L Volkmer JP George BM. Anti-SIRPα antibody immunotherapy enhances neutrophil and macrophage antitumor activity. Proc Natl Acad Sci USA. (2017) 114:E10578–e85. 10.1073/pnas.1710877114 29158380 PMC5724266

[97] Murata Y Tanaka D Hazama D Yanagita T Saito Y Kotani T. Anti-human SIRPα antibody is a new tool for cancer immunotherapy. Cancer Sci. (2018) 109:1300–8. 10.1111/cas.13548 29473266 PMC5980332

[98] Yanagita T Murata Y Tanaka D Motegi SI Arai E Daniwijaya EW. Anti-SIRPα antibodies as a potential new tool for cancer immunotherapy. JCI Insight. (2017) 2:e89140. 10.1172/jci.insight.89140 28097229 PMC5214103

[99] Koh E Lee EJ Nam GH Hong Y Cho E Yang Y. Exosome-SIRPα, a CD47 blockade increases cancer cell phagocytosis. Biomaterials. (2017) 121:121–129. 10.1016/j.biomaterials.2017.01.004 28086180

[100] Cai B Dongiovanni P Corey KE Wang X Shmarakov IO Zheng Z. Macrophage MerTK promotes liver fibrosis in nonalcoholic steatohepatitis. Cell Metab. (2020) 31:406–21.e7. 10.1016/j.cmet.2019.11.013 31839486 PMC7004886

[101] Hua L Shi J Shultz LD Ren G. Genetic models of macrophage depletion. Methods Mol Biol. (2018) 1784:243–58. 10.1007/978-1-4939-7837-3_22 29761404 PMC6333569

[102] Moreno SG. Depleting macrophages in vivo with clodronate-liposomes. Methods Mol Biol. (2018) 1784:259–62. 10.1007/978-1-4939-7837-3_23 29761405

[103] Ries CH Cannarile MA Hoves S Benz J Wartha K Runza V. Targeting tumor-associated macrophages with anti-CSF-1R antibody reveals a strategy for cancer therapy. Cancer Cell. (2014) 25:846–59. 10.1016/j.ccr.2014.05.016 24898549

[104] Duffield JS Forbes SJ Constandinou CM Clay S Partolina M Vuthoori S. Selective depletion of macrophages reveals distinct, opposing roles during liver injury and repair. J Clin Invest. (2005) 115:56–65. 10.1172/JCI200522675 15630444 PMC539199

[105] Best J Verhulst S Syn W-K Lagaisse K van Hul N Heindryckx F. Macrophage depletion attenuates extracellular matrix deposition and ductular reaction in a mouse model of chronic cholangiopathies. PLoS ONE. (2016) 11:e0162286. 10.1371/journal.pone.0162286 27618307 PMC5019458

[106] Bader JE Velazquez KT Enos RT Carson MS Cranford TL Nagarkatti P. Macrophage depletion decreases tumorigenesis and alters gut microbiota in the AOM/DSS mouse model of colon cancer. J Immunol. (2017) 198(1 Supplement):66. 10.1152/ajpgi.00229.2017 29025731 PMC5866374

[107] Fritz JM Tennis MA Orlicky DJ Lin H Ju C Redente EF. Depletion of tumor-associated macrophages slows the growth of chemically induced mouse lung adenocarcinomas. Front Immunol. (2014) 5:587. 10.3389/fimmu.2014.00587 25505466 PMC4243558

[108] Cailhier JF Partolina M Vuthoori S Wu S Ko K Watson S. Conditional macrophage ablation demonstrates that resident macrophages initiate acute peritoneal inflammation. J Immunol. (2005) 174:2336–42. 10.4049/jimmunol.174.4.2336 15699170

